# The Analysis of Environmental Cost Control of Manufacturing Enterprises Using Deep Learning Optimization Algorithm and Internet of Things

**DOI:** 10.1155/2022/1721157

**Published:** 2022-09-30

**Authors:** Jin Qiu, Wenzhuo Chen

**Affiliations:** ^1^Guangdong University of Science and Technology, Dongguan 523000, China; ^2^Department of Electronics and Information Engineering, North China Institute of Science and Technology, Langfang 065201, China

## Abstract

Under the background of the Internet of things (IoT), the problems between the actual production and the environment are also prominent. The environmental cost control in the production process of manufacturing enterprises are discussed to reduce the environmental cost and promote the improvement of production efficiency. First, the environmental cost under the background of IoT is analyzed. Also, the environmental cost control methods in the production process of traditional manufacturing enterprises are investigated. Second, based on the principle of traditional genetic algorithm, the fast-nondominated sorting genetic algorithm (NSGA-II) of multiobjective genetic algorithm is introduced to complete the optimization of BP neural network (BPNN) algorithm in deep learning (DL), and the multiobjective GA optimization BPNN model is established. Finally, the multiobjective GA algorithm is used to empirically analyze the environmental cost control capability of a paper-making enterprise. It is compared with enterprises with excellent and poor environmental cost control capabilities in the same industry to find out secondary indexes. The results show that environmental costs have long-term and economic characteristics. The global search ability of BPNN optimized by multiobjective GA is improved, and the local optimal dilemma is avoided. Through empirical analysis, it is found that the comprehensive capability of the environmental cost control of the enterprise is better, scored 79 or more, and the indexes of insufficient development and advantages are obtained. As IoT rapidly develops, it is necessary to further improve the ability of enterprises in environmental cost management, which is very important to promote the development of enterprises and enhance their core competitiveness. It is hoped that this investigation can provide certain reference significance for improving the environmental cost management capability of enterprises, increasing production efficiency, and reducing environmental costs.

## 1. Introduction

The soaring economic growth in China has seen the explosion of manufacturing enterprises, which has brought greater pressure on the ecological environment. Currently, the environmental cost is an important index for the development and production of the green economy and plays a significant role in promoting economic development in China. The environmental cost is first proposed as early as the 20th century based on the analysis of the relationship between economic development and environmental protection [1]. Scholars worldwide have explored the classification of environmental costs, which is discussed from the scope of the enterprise's production activities, the main body of responsibility, as well as the utilization and consumption of resources. Then, numerous classification methods are proposed. With social progression, the concept and classification of environmental costs have been perfected [2]. With further analysis of environmental costs, the control methods of environmental costs are constantly increasing. Some scholars have analyzed the main drivers of environmental cost management. The main components of environmental cost drivers are the work process and the organizational structure of the enterprise. Therefore, a management and analysis system should be established for enterprise environmental cost management [3]. Some scholars believe that the management of environmental costs of the enterprise should start from the perspective of advanced control. Thus, preventive management should be carried out to reduce the environmental cost, and enterprises are managed and controlled from the source of environmental pollution [4]. Other scholars have determined the environmental cost through the cost accounting concept in the comprehensive environmental and economic accounting management system. Then, it is combined with the ecological footprint model to account for the environmental cost caused by the occupation of ecological resources [5]. At present, scholars worldwide have conducted an in-depth investigation of the management and control methods of the environmental cost. Besides, they have explored the management and control of the environmental cost through various methods.

Afterward, many scholars have evaluated the environmental cost control capability. Some scholars have analyzed the links and factors of enterprise environmental cost control using the fuzzy analytic hierarchy process so that the enterprise can observe the advantages and disadvantages of the application of environmental cost control. To achieve sound and rapid development, an enterprise should recognize its current level of development in the same industry. The same is true for improving environmental cost control capability. In recent years, with the development of DL (Deep Learning) technology, more scholars have applied DL technology to evaluate enterprise development while achieving good results [6]. Here, DL technology and the GA (Genetic Algorithm) are combined to evaluate the environmental cost control capability of the enterprise.

Based on the existing results, the method of environmental cost control of manufacturing enterprises is explored. Meanwhile, related concepts, such as environmental costs of the enterprises, are analyzed. Then, the BP (Backpropagation) NN (Neural Network) algorithm is optimized through the multiobjective GA and DL technology. At the same time, the analytic hierarchy process is combined to evaluate the environmental cost control indexes of the enterprises. The multiobjective GA optimization is used for the index evaluation of insufficient environmental cost control capabilities of the enterprises.

## 2. Methods

### 2.1. Environmental Cost Control of Enterprises

Environmental costs have long-term development, and economic environmental costs include two parts: control cost and damage cost. It is the cost incurred by the enterprise's environmental protection measures to comply with environmentally friendly principles. Also, it includes other costs incurred to protect the environment [7]. Enterprises should take the initiative to protect the environment. In the process of production and operation activities, to achieve the purpose of protecting the environment, the activities costs, such as preventive expenditure, maintenance expenditure, and governance expenditure, all belong to the category of the enterprise environment. There are various types of environmental costs of enterprises. According to the period of the production and operation activities that the enterprise is engaged in, it mainly includes three costs before, during, and after the activity [8]. As the name implies, the ex ante cost refers to the cost incurred by enterprises to protect the environment before carrying out production activities. It includes the cost of improving the production process, introducing environmental protection equipment, and training employees on environmental protection knowledge. It is beneficial to reduce the cost of enterprises in production activities. The cost in the process of activity refers to the cost incurred by the enterprises to protect the environment during the production activities. It includes maintenance costs of environmental protection equipment and costs incurred in environmental testing. The ex post cost mainly refers to the cost incurred by the enterprise in the treatment and repair of the environmental pollution generated by itself. The main purpose is to make the environment self-belief and enhance the self-recovery ability.

*Environmental Cost Control*. Environmental cost control refers to the process by which enterprises control and manage environmental costs through scientific means, thereby predicting, calculating, evaluating, and analyzing environmental costs in an organized manner. According to the requirements of relevant national laws and regulations, enterprises can achieve the goal of green and sustainable operation [9]. Environmental costs are different from the general costs of enterprises. The environmental costs of enterprises cannot be blindly reduced. When managing the environmental cost of an enterprise, the social benefits it brings must be considered. When reducing the environmental cost of an enterprise, it is necessary to consider how to increase the individual benefits of the enterprise as well as ensure the safety and quality of the ecological environment. When conducting environmental cost management of an enterprise, one should not only pursue its operating profit one-sidedly. It is necessary to take the concept of sustainable development as its green business philosophy, thereby effectively managing the environmental costs of enterprises in real time and reducing the pollution degree of the environment caused by enterprises when they are engaged in business activities as much as possible. Enterprises need to pay attention to environmental protection while pursuing operating profits. It makes the social and economic benefits of the enterprise reach a balanced and unified state, thereby laying a solid foundation for the long-term green and healthy development of the enterprise [10].

*Characteristics of Environmental Cost Control*. It refers to the need for a relatively long development stage when the enterprise controls and manages environmental costs, thereby completing the management and control of the environmental costs step by step. In this process, enterprises need to pay attention to the requirements for environmental cost control at the current stage. Moreover, it is necessary to predict the environmental costs that the enterprises may incur in the future and take measures in advance. In the initial stage, when enterprises control environmental costs, the manpower, material resources, and economic costs required to invest are relatively high. At this stage, the cost pressure of environmental management faced by enterprises is relatively high. However, with the continuous development of the enterprises, the cost invested in the initial stage may save many economic resources for later development. It is also conducive to the growth of the enterprises' core competitiveness. Economy refers to that the control of environmental costs by enterprises is to maximize the economic efficiency, maximize the benefits of resource utilization, and reduce the pollution of the enterprises to reduce the environmental protection expenditures. The input structure of the enterprises' environmental costs is optimized to reduce the cost pressure, keep the environmental costs at a reasonable level, and maximize the economic benefits of the enterprises [11]. The long-term characteristics of environmental costs are reflected in the development of enterprises. Therefore, starting from the economy of the environmental costs of enterprises, the control of environmental costs is analyzed and explored.

### 2.2. Multiobjective GA Optimization

There are two main methods for solving multiobjective optimization problems: traditional optimization algorithms and intelligent optimization algorithms. Most of the traditional optimization algorithms convert multiple objectives that need to be optimized into one objective and optimize the solution through a single-objective optimization method. Commonly used methods include the weighting method and the objective planning method. Currently, commonly used intelligent optimization algorithms include GA and PSO (Particle Swarm Optimization) algorithms [12]. Among them, the GA is a global optimization algorithm that searches randomly. By simulating the evolutionary laws of genetic selection and survival of the fittest, the solution of the problem is regarded as a population. The fitness of the solution is used as the basis for judging whether the solution is excellent. The excellent solution is inherited to the next generation, approaching the optimal solution through continuous evolution from generation to generation [13].

When calculating with the GA, it is necessary to pay attention to the calculation of fitness and the determination of the coding method. Among them, fitness is the basis for judging the pros and cons of individuals in a population. Excellent individuals are selected for inheritance through fitness. Then, in the calculation process, the solution of the real problem cannot be directly calculated by the GA. It needs to be encoded and transformed into chromosomes into a form that can be calculated by the GA. This algorithm is simple to operate, with strong versatility and global search capabilities [14].

**Figure 1:**
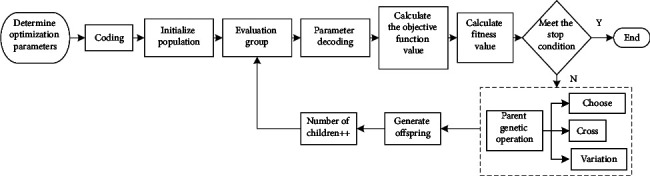
The calculation process of the GA.

The process of the GA is shown in Figure 1.

When calculating with the GA, it must first be coded. The process of composing the solution of the problem into a chromosome in a certain order is coding. Conversely, the process of translating the best individual chromosomes obtained by the GA into the solution of the problem is decoding. Currently, the commonly used chromosome coding methods include binary coding, parameterized coding, and direct coding [15].

*Initializing the Population*. The GA is an algorithm that evolves the population operation. In the initial stage of calculation using the algorithm, an initial population needs to be generated, which may affect the final result.

*Fitness Calculation*. When the GA is performing genetic operations, the selection of the genetic population is judged based on the fitness value of individuals in the population. The higher the fitness value of an individual, the better the individual. The fitness value of an individual is generally calculated by the objective function of the optimization problem. In the process of this investigation, the objective function to be calculated belongs to the problem of minimization. When transforming it into a fitness function, there are generally two methods of transforming it into an opposite number and a derivative. The transformation method is as follows [16].

(1) $$\begin{array}{c} fitnessfun\;c\left(x\right)=-f\left(x\right)\text{,} \end{array}$$

(2) $$\begin{array}{c} fitnessfun\;c\left(x\right)=\frac{1}{f\left(x\right)+e}\text{.} \end{array}$$

Here, *f*(*x*) represents the minimum objective function that is always nonnegative. *e* represents a greatly small number to prevent the denominator from being zero.

*Genetic Operation*. The selection operation is used to determine which good individuals can be used as parents to reproduce the next generation. Generally, individuals with high individual fitness values in the population will be retained. Currently, commonly used selection methods include the roulette selection method, random traversal sampling method, tournament selection method, and index sort selection method [17]. The crossover operation will recombine part of the chromosomes of each individual in the parent to produce offspring that combines the information of the parent. The crossover operation may make the generated offspring have higher fitness. Currently, the commonly used crossover operation methods include single-point crossover, two-point crossover, multipoint crossover, and mixed uniform crossover [18]. The mutation operation is to change the genes on the chromosomes of the offspring with a small probability, so that the individuals of the population have diversity, to prevent the population from premature convergence. At the same time, it allows the GA to have the ability of local random search to avoid the occurrence of local optimal phenomena. The method of mutation operation is related to the method of coding. Currently, the commonly used methods include binary mutation, real number mutation, and Gaussian mutation [19].

*Parameter Adjustment*. The GA mainly involves parameters such as population size, crossover probability, mutation probability, evolutionary algebra, and population initialization method. These parameters are used to determine whether the population will be precocious [20]. The population size will affect the calculation efficiency of the algorithm. The population that is too small will cause diseased genes in the offspring, and too large one will cause the calculation efficiency of the algorithm to decrease. Thus, the population size is usually selected in the range of 100 to 200. The value range of the crossover probability is generally between 0.4 and 0.99. If the mutation probability is too large, the good genes in the population will be destroyed. If it is too small, the diversity of the population will decrease, and the algorithm will easily fall into the trouble of local optimization. Generally, the range of mutation probability is set at 0.00001 to 0.2. Evolutionary algebra will affect the convergence times of the algorithm. Too large will cause the subsequent calculations of the algorithm to be meaningless. Too small will cause the algorithm to converge prematurely, which may cause the algorithm to fail to obtain the true optimal solution. The initialization of the population will determine the number of iterations of the algorithm to a large extent. Therefore, it is necessary to estimate the calculation result during initialization and initialize the algorithm near the global optimal solution.

GA has a strong global search: GA can improve its solving efficiency through parallel computing when facing large-scale or multiobjective problems. The mutation and crossover operations are used to prevent the algorithm from falling into a local optimal state. *Strong Versatility and Controllability*. GA can adjust its coding and genetic operations according to the actual situation and change its mutation and crossover operations so that it has strong controllability and versatility. *Good Scalability and Upgradeability*. The operating steps of the GA are simple, and it can be combined with other algorithms to make up for the deficiencies of the algorithm itself, which can be better applied to practical problems. The GA is self-organizing, adaptive, and self-learning [21].

### 2.3. Multiobjective GA Optimization

**Figure 2:**
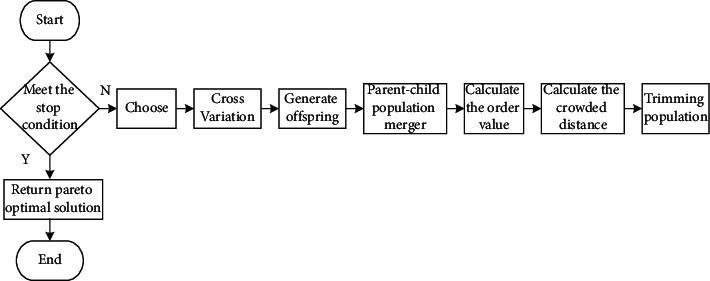
The calculation process of the NSGA-II algorithm.

In environmental cost control of enterprises, due to the need to consider multiple optimization objectives, traditional GA cannot solve multiobjective problems well. The multiobjective optimization problem is mainly to ensure that satisfactory solutions can be obtained for multiple optimization objectives [22]. Therefore, multiobjective GA optimization is selected to control the environmental cost of enterprises. In the process of selection, the population is stratified through the dominance relationship among the population. The set of individuals that are not dominated is called the Pareto solution set. The probability of this solution set is passed to the next generation is greater. In the multiobjective GA, it is necessary to find the solution of Pareto and find the optimal solution in the solution set [23]. The fast NSGA-II (Nondominated Sorting GA) with elite strategy in the multiobjective GA is selected to explore the environmental cost control of enterprises. The multiobjective GA runs fast, and the solution set has good convergence. At the same time, the complexity of the NSGA-II is lower [24]. The calculation process of this algorithm is shown in Figure 2.

DL technology has been widely used in the field of computer vision research. Meanwhile, it has an excellent performance in image and time-series data processing. Currently, there are feedforward NN, RNN (Recurrent Neural Network), and self-organizing NN under DL technology [25].

BPNN is a typical multilayer feedforward NN, which mainly relies on the BP of errors during training. The weights and thresholds of the network are adjusted through BP, thus solving the nonlinear problem. The learning process of the BPNN can be divided into two stages of forwarding propagation and backward propagation [26]. The typical BPNN structure includes an input layer, hidden layer, and output layer. The signal is forwarded layer by layer in the BPNN until the output layer. Therefore, the NN is a parallel multilayer feedforward network [27].

**Figure 3:**
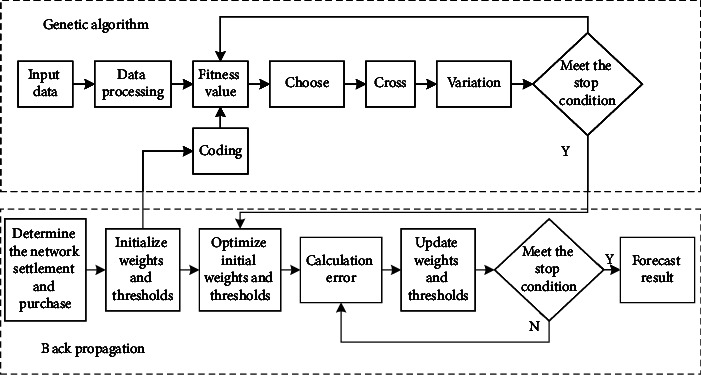
The process of the GA-BP model.

*Construction of Multiobjective GA Optimization*. BPNN is optimized through the multiobjective GA, and the GA-BP (multiobjective GA optimization BPNN) model is constructed. The constructed GA-BP model includes three parts: the establishment of the BPNN model, the optimization of BPNN with GA, and the prediction of optimized BPNN [28]. The process of the GA-BP model is shown in Figure 3.

### 2.4. Evaluation of Environmental Cost Control of Enterprises Based on the GA-BP

The NN relies on plenty of sample data for training and performs pattern recognition based on the features of the sample. The input feature vector, network weight, and threshold value in the NN jointly determine the output. Thus, the choice of the feature vector is greatly important [29]. The input data for NN training should be distributed with spatial continuity. Sample data with correlated features should be selected for input to reduce the similarity of the samples and keep the data dimension consistent.

The GA-BP model is established on the MATLAB platform, and coding operations are performed. The input dimension of the network is determined by the number of feature vectors. The input network has the highest probability in each mode, which is the recognized mode [30]. The feature vector is compared with the feature vector of poor or excellent samples in the same industry. The features with larger gaps are taken as guidance data. The NN selects data through random sampling and optimizes it by orthogonal experiment [31]. The networks under different parameters are screened. The GA is mainly to initialize the weights and thresholds of the NN after screening.

In manufacturing enterprises, different types of manufacturing systems have great differences in basic information. The GA-BP model can evaluate and optimize the environmental cost control capability of manufacturing enterprises. A paper-making enterprise is taken as the research object to analyze its environmental cost control capability. First, the indexes and weights of environmental cost control are determined using the analytic hierarchy process [32]. Through the analysis of the actual operation of the enterprise, based on the principle of relevance and comprehensiveness, the environmental control indexes of the enterprise are divided into 4 primary indexes and 16 secondary indexes. There is a corresponding relationship between the primary indexes and secondary indexes. The determined evaluation indexes and corresponding numbers are shown in Table 1.

The analytic hierarchy process combined with the fuzzy comprehensive evaluation method is used for index evaluation, thereby getting the final weight of the index. The comprehensive membership degree, comprehensive score, and index score rate are calculated.

After each piece of data is calculated, the data are transformed. Through data discretization and normalization processing, the feature vector of each data index is obtained [33]. The feature vector data are input into the trained GA-BP model [34–37]. The gap of the environmental cost control capability between the paper-making enterprise and the same type of enterprise is obtained. The enterprise's scoring results in the entire industry are obtained. Then, the corresponding poor samples under its primary indexes are selected and analyzed [38–40]. Meanwhile, the control scheme of the IoT platform is adopted to ensure the ease of operation and multiterminal compatibility of enterprises in environmental control [41–43]. Specifically, the Socket-Websocket bridging module is employed to ensure the real-time of IoT so that the Web application in the ordinary browser can control the environmental monitoring equipment in real time, like industrial control software [44–48].

## 3. Results and Analysis

### 3.1. Index Weight Analysis

**Figure 4:**
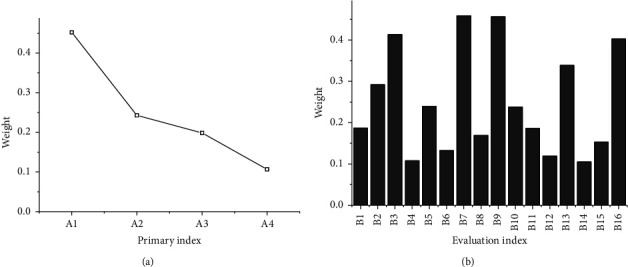
The calculation results of the weights of each index. (a) The calculation results of the weights of the primary indexes. (b) The calculation results of the weights of the secondary indexes

Through the analytic hierarchy process combined with the fuzzy comprehensive evaluation method, the weight of each index is obtained. The results are shown in Figure 4.

Figure 4 illustrates that among the primary indexes, the internal input index of environmental protection *A*1 has the highest weight, indicating that internal input has a relatively large impact on the environmental cost control capability of the enterprise. Among the secondary indexes, the indexes, such as the update of environmental protection technology and equipment *B*3, pollutant emission reduction *B*7, comprehensive utilization rate of waste *B*9, and the review situation of environmental protection department *B*16, have relatively high weight values. The results indicate that these indexes have a relatively large impact on the primary indexes.

### 3.2. Analysis and Calculation Results of Comprehensive Membership Degree

**Figure 5:**
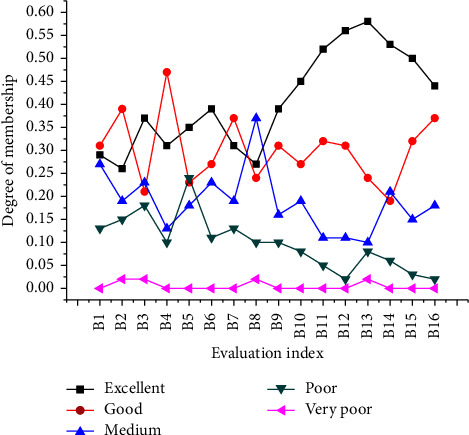
Analysis results of the membership degree of each index.

The calculation of the comprehensive membership degree is obtained through a QS (Questionnaire Survey). A total of 150 environmental QSs are issued. Then, 120 valid QSs are recovered, with a valid rate of 80%. The subjects of the QS include, but are not limited to, internal personnel of the enterprise related to environmental protection. According to the scores, the survey results are divided into five criteria: excellent, good, medium, poor, and very poor. The sum of the survey results of excellent, good, and medium is used as the evaluation criterion for the degree of approval of the index table. The sum of the evaluation results of poor and very poor is used as the basis for calculating the degree of disapproval. The final results of calculating the membership degree of each index are shown in Figure 5.

Figure 5 shows that the paper-making enterprises do a better job in controlling the environmental cost in production. Moreover, the evaluation recognition of each index is high. Among them, the membership degree of expenditure on purchasing environmental protection raw materials and updating environmental protection technology and equipment is relatively poor. About 20% of the employees are not in favor of purchasing environmental protection raw materials and updating environmental protection technology and equipment, and the degree of nonrecognition of green product R&D (Research and Development) expenses is more than 15%. The results show that enterprises should increase investment in R&D of green products to improve the level of R&D.

### 3.3. An Empirical Test on the Cost of Enterprise Environmental Loss

**Figure 6:**
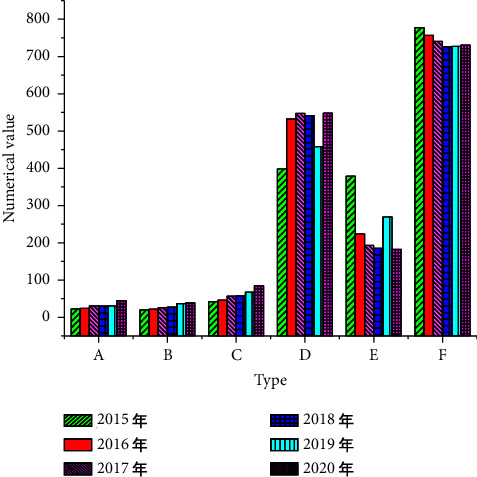
Environmental cost of *X* coal enterprise in 2015–2020 (A: environmental prevention cost, B: environmental detection cost, C: environmental control cost, D: environmental internal loss cost, E: environmental external loss cost, and F: environmental loss cost).

Figure 6 below shows the environmental cost details of *X* coal enterprise from 2015 to 2020.

**Figure 7:**
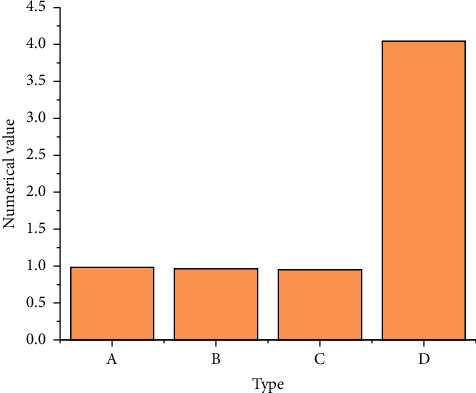
Loss cost priority analysis (A: *R*; B: *R*^2^; C: adjusted *R*^2^; and D: standard skewness error).

Figure 7 shows the loss cost priority analysis based on Figure 6.

**Figure 8:**
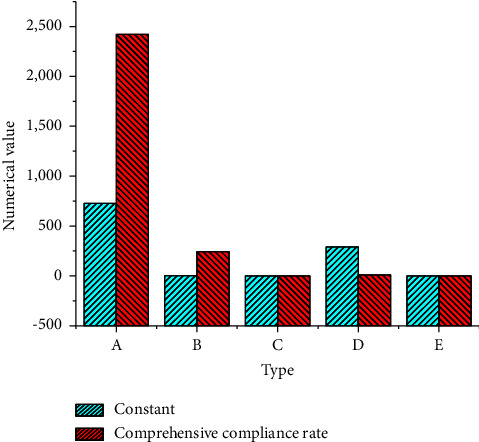
Test results of constant term and coefficient (A: *B*-value; B: standard error; C: beta value; D: *T*-value; and E: significance).

Correlation coefficient *R* = 0.983, judgment coefficient *R*^2^ = 0.966, adjusted *R*^2^ = 0.951, and standard error of regression estimation *s* = 4.046, indicating that the regression effect of this group of data is general. The test results of constant term and coefficient are shown in Figure 8.

Figure 8 displays that when the environmental control cost and the environmental loss cost function are equal, the specific value of the target rate can be obtained, but this value is somewhat idealized. This value is the minimum value of the total environmental cost function, but it is not necessarily the lowest point of the enterprise's environmental cost, which must be determined according to the actual situation of *X* coal enterprise.

### 3.4. Analysis of Environmental Cost Control Capability of Enterprises Based on the GA Optimization

**Figure 9:**
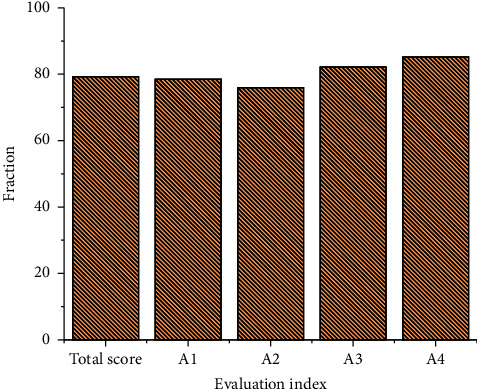
Analysis results of environmental cost control capability of the enterprise based on the genetic optimization algorithm.

A general evaluation is made on the environmental cost control capability of the enterprise. The primary index of the environmental control capability of the enterprise is evaluated.  Figure 9 shows the statistical results of the evaluation.

As can be seen from the above figure, the total score of the environmental cost control capability of the enterprise is above 79. In general, the environmental cost control capability of the enterprise is relatively good. From the primary evaluation index, the score of the cleaner production index is the lowest, only reaching 75%. But relatively speaking, the score of this index is relatively high. In the index scores of this level, the scores of the pollution control effect index and the external control effect index are both above 80%. It shows that these two measures are implemented relatively well in the environmental cost control of the enterprise. However, in the actual environmental protection process, further maintenance and improvement are needed to strive to improve the enterprise's ability to control environmental costs. After calculation, the *p*-value of these two variables is 0.01, which is less than 0.05, so the research on pollution control effect index and external control effect index has statistical significance. At the same time, following the analysis of these two variables, the control effect of enterprises on environmental cost is further understood to formulate relevant strategies to improve the enterprise's environmental cost control capability.

### 3.5. Comparison with Other Samples in the Same Industry

The multiobjective GA optimization is used to compare the environmental cost control index of this paper-making enterprise with the feature vector of the poor and excellent enterprises in the same industry. Through the search of the algorithm, Table 2 shows the statistics of the analysis results of the secondary indexes with the largest sample gap characteristics under each primary index.

As can be seen from the above table, when compared with excellent enterprises, the secondary indexes with the largest gap between this enterprise and excellent enterprises are the update of environmental protection technology and equipment, pollutant emission reduction, the compliance rate of pollutant discharge, and environmental protection tax and pollution fine. It shows that the enterprise needs to focus on improving these sample features in its subsequent development. When compared with the features of poor enterprises, the enterprise performs well in several aspects, such as research and development fees of green products, energy-saving, the number of accidental pollution incidents, and environmental complaints. The enterprise needs to continue to maintain the advantages of these indexes in the subsequent development process. At the same time, it should improve its deficiencies and approach the standard of environmental cost control of excellent enterprises. Moreover, the enterprise needs to improve its environmental cost control capability, while maintaining a steady rise in its core competitiveness.

## 4. Conclusion

The environmental cost control of manufacturing enterprises in the development process is mainly explored. Through the analysis of environmental costs and other related concepts, it is found that environmental costs have long-term and economic characteristics. Therefore, it is necessary to pay attention to the management and control of environmental costs in the development process to lay a solid foundation for the development of enterprises. The principle of traditional GA is analyzed. The multiobjective GA is used to improve the BPNN, which can improve the global search ability of the BPNN, avoid the dilemma of local optimization, and improve the algorithm's computing performance. Finally, the multiobjective GA optimization is used to empirically analyze the environmental cost control ability of a paper-making enterprise. It is found that the comprehensive score of the environmental cost control capability of the enterprise is above 79. It is proved that the environmental cost control capability of the enterprise is better while obtaining indexes of insufficient development and advantages. It is necessary to further improve the environmental cost management capability of the enterprise to promote the core competitiveness of its development. At the same time, the simulation analysis indicates that the total score of enterprise environmental cost control capability is more than 79, and the overall enterprise environmental cost control capability is better. In terms of the primary evaluation index, the score of the cleaner production index is the lowest, only 75%. But the score of this index is relatively high. Compared with the same level of indexes, the scores of the pollution control index and external control index are more than 80%.

Still, there are some shortcomings; the focus is on the analysis of the principles of the research methods. Due to space limitations, the BPNN and modeling methods are briefly introduced. The investigation is more inclined to theoretical analysis and lacks certain practice. It is hoped that, in the follow-up, this model method can be applied to plenty of practical investigations to further verify the performance of the proposed model.

## Figures and Tables

**Table 1:** Evaluation indexes of environmental cost control of enterprises based on the GA-BP model.

	Primary index (*A*)	Secondary index (*B*)
Evaluation indexes of environmental cost control capability of enterprises	Internal input index of environmental protection *A*1	Environmental training education fee of employees *B*1
		The research and development fee of green products *B*2
		The update of environmental protection technology and equipment *B*3
		Environmental protection system construction, department establishment *B*4
	Cleaner production index *A*2	Expenditure on purchasing environmentally friendly raw materials *B*5
		Energy-saving *B*6
		Pollutant emission reduction *B*7
		Hazardous waste storage expenditure *B*8
	Pollution control effect index *A*3	The comprehensive utilization rate of waste *B*9
		The compliance rate of pollutant discharge *B*10
		Staff health status *B*11
		Number of accidental pollution incidents *B*12
	External influence index *A*4	Environmental protection tax and pollution fine *B*13
		Environmental information disclosure of enterprises *B*14
		Environmental complaints *B*15
		The review situation of environmental protection department *B*16

**Table 2:** Analysis results of the secondary indexes with the largest sample gap feature under each primary index.

Primary index	Secondary index (excellent sample feature)	Secondary index (poor sample feature)
Internal input index of environmental protection *A*1	The update of environmental protection technology and equipment *B*3	The research and development fee of green products *B*2
Cleaner production index *A*2	Pollutant emission reduction *B*7	Energy-saving *B*6
Pollution control effect index *A*3	The compliance rate of pollutant discharge *B*10	Number of accidental pollution incidents *B*12
External influence index *A*4	Environmental protection tax and pollution fine *B*13	Environmental complaints *B*15

## Data Availability

The raw data supporting the conclusions of this study can be obtained from the corresponding author upon request.
